# Radiation-induced bystander effect and its clinical implications

**DOI:** 10.3389/fonc.2023.1124412

**Published:** 2023-04-05

**Authors:** Haoyi Tang, Luwei Cai, Xiangyang He, Zihe Niu, Haitong Huang, Wentao Hu, Huahui Bian, Hao Huang

**Affiliations:** ^1^ State Key Laboratory of Radiation Medicine and Protection, School of Radiation Medicine and Protection, Collaborative Innovation Center of Radiological Medicine of Jiangsu Higher Education Institutions, Soochow University, Suzhou, China; ^2^ Nuclear and Radiation Incident Medical Emergency Office, The Second Affiliated Hospital of Soochow University, Suzhou, China

**Keywords:** radiation, DNA damage, LNT, non-targeted effects, bystander effects

## Abstract

For many years, targeted DNA damage caused by radiation has been considered the main cause of various biological effects. Based on this paradigm, any small amount of radiation is harmful to the organism. Epidemiological studies of Japanese atomic bomb survivors have proposed the linear-non-threshold model as the dominant standard in the field of radiation protection. However, there is increasing evidence that the linear-non-threshold model is not fully applicable to the biological effects caused by low dose radiation, and theories related to low dose radiation require further investigation. In addition to the cell damage caused by direct exposure, non-targeted effects, which are sometimes referred to as bystander effects, abscopal effects, genetic instability, etc., are another kind of significant effect related to low dose radiation. An understanding of this phenomenon is crucial for both basic biomedical research and clinical application. This article reviews recent studies on the bystander effect and summarizes the key findings in the field. Additionally, it offers a cross-sectional comparison of bystander effects caused by various radiation sources in different cell types, as well as an in-depth analysis of studies on the potential biological mechanisms of bystander effects. This review aims to present valuable information and provide new insights on the bystander effect to enlighten both radiobiologists and clinical radiologists searching for new ways to improve clinical treatments.

## 1 Introduction

Radiation is a known carcinogen that is prevalent in all aspects of daily life, and low dose radiation is present everywhere and is used extensively in nuclear technology, medical examinations, as well as in other fields. In 1986, the United Nations Scientific Committee on the Effects of Atomic Radiation (UNSCERA) stipulated (1) that low level radiation refers to low linear energy transfer (LET) radiation at doses of less than 0.2 Gy or high LET radiation at doses of less than 0.05 Gy, provided that the dose rate is within 0.05 mGy/min. Currently, low dose radiation refers to exposure doses that match the aforementioned requirements and have a dose rate greater than 0.05 mGy/min. In contrast, according to the National Academy of Sciences Research Council’s BEIR-VII Phase II report on the Biological Effects of Ionizing Radiation published in 2006, low dose radiation is defined as radiation at doses of 100 mSv or less and dose rates of less than 0.1 mSv/min. Although the definition of low dose radiation varies from one institution to another, the general differences are not significant, and the relevant regulations can be applied depending on the circumstances.

It is known that the Chernobyl incident in 1986 had a lasting impact on the surrounding environment. Since most of the population had evacuated the area after the accident, it is somewhat difficult to understand the effects of low-dose radiation on the long-term effects on humans in the surrounding area. Thus, studies have been carried out on large mammals (dogs) in the area of the accident and have uncovered important effects of long-term low-dose radiation in animal genetic events, such as the sharing of haplotypes likely contribute to differentiation, which has contributed to our understanding of the processing of radiation events and the biological mechanisms behind them (2). Because radiation-related phenomena have been studied in detail, there have been general agreement over the past few decades on the paradigm that radiation causes biologically relevant responses by damaging DNA through direct action on the nuclei of cells, which can be called the ‘‘targeted effect’’. In simple terms, it was thought that the damage caused by radiation to the organism was based on radiation-nucleus interactions. However, with the development of microbeam technology, scientists have been able to target radiation to the cytoplasm, and in subsequent investigations, there have been concrete evidence that cells manifest similar changes even when radiation is not targeted directly at the nucleus. Furthermore, the adjacent unirradiated cells may receive signals from the irradiated cells and produce a damage response. This type of response in unirradiated cells or organisms that resemble irradiated cells is known as “non-targeted effects” (NTEs) (3–5).

In general, the guidelines on radiation protection mainly follow the linear no-threshold (LNT) model. Based on long-term observations of Japanese atomic bomb survivors, the International Commission on Radiological Protection and the United States National Council on Radiation Protection and Measurements recommend using this data set to evaluate the risks associated with radiation at doses above 50 mSv, i.e. using the LNT model. The model contends that there is no so-called “threshold” and that the risk of radiation-induced cancer rises with dose and the risk exists even at low doses. This model is in line with the conventional understanding of radiation that even a low dose of radiation can increase the risk of disease in humans. However, the paradigm has been challenged in recent decades due to the further study of extra-nuclear and extracellular radiation events. In addition, there is growing evidence (6–8) that the LNT model may not be appropriate for use in the low dose field.

Bystander effects, abscopal effects, and genetic instability are the three primary categories of NTE. This review will focus on the bystander effect, and make an effort to evaluate how it might be applied in clinical settings in the future.

Precise parameters and relatively comprehensive models are essential if bystander effects are to be accurately described. With the help of mathematical models, we can compare different candidate signaling molecules and pathways with experimental data, assess the degree of fit between them, and filter them for the prediction of bystander effects in realistic biological systems where conditions are more complex and less easily controlled. Most of the proposed biophysical models (9–17) differ in the fundamental requirements and conditions. They basically fix some parameters or rely only on a single reference system for the analysis of the experimental results to produce a model that is simple to implement and easy to handle, while in reality, the fixation of these parameters is often difficult to achieve. A key reason for preventing the general applicability of many models is that the interactions between variables in the bystander effect are not clear, for instance, is the signal generated by the bystander effect radiation quality dependent? Does the bystander effect vary according to the type of cells that are exposed to radiation? These problems pose a challenge to the current models, leading to a number of models that have been verified under particular circumstances but lack universal applicability. Furthermore, the distortions and inaccuracies in the results caused by various interferences can be diminished if some weighting is given to bystander effects, cohort effects, and abscopal effects induced by low dose radiation.

With a deeper understanding of the bystander effect, various biophysical models have been proposed. Although a lot of recent research has concentrated on the molecular mechanisms underlying the bystander effect in humans or other complex systems, or the confirmation of these effects in these systems, the guidance for particular therapeutic practices remains limited. Nevertheless, treatment techniques that use the bystander effect can be anticipated in the clinical arena if future research in both fields is unified and breakthroughs are achieved.

## 2 Background of the bystander effect

Radiation-induced bystander effect (RIBE) proposes that signals generated from irradiated cells induce a similar effect in unirradiated cells as in the irradiated cells. The so-called bystander effect was first discovered in 1992 by Nagasawa and Little, who used 0.31 mGy alpha particles to irradiate Chinese hamster ovary (CHO) cells and found that although less than 1% of the nuclei were traversed by alpha particles, approximately 30% of the cells showed an increased frequency of sister chromatid exchange. Contrarily, under normal circumstances, a radiation dose of approximately 2.0 Gy would be required to produce a comparable radiobiological effect (18).

Murphy and Morton described a phenomenon called the “bystander effect” in 1915 (19). Using tumor transplantation experiments, they found that approximately 50% of mice exhibited tumor immunity after irradiation due to the “bystander effect”. They hypothesized that exposure of normal tissue to irradiation could affect tumor growth and explained the phenomenon relative to their previous hypothesis of “lymphoid elements”. Gene mutation studies had not started when the Murphy experiments were completed, hence a definite connection between radiation exposure and chromosomal damage was not demonstrated at that time. The “target model” was not established until the 1930s, when Muller’s mutation experiments (20) were conducted and combined with the related work of Timofeev-Ressovsky (1936), Zimmer (1936), and Delbruck (1940). In the 1940s, the indirect effects of radiation were correlated with free radical formation, and although it can explain the indirect effects of radiation, the effects of free radicals have proven to be secondary and transient. Thereafter, researchers mostly considered DNA as the key target, and the “signaling molecules” were not identified because of the inability to establish mechanisms of action. It was not until the late 1980s to early 1990s that this paradigm changed, shifting from the study of DNA to signal regulation and tissue responses. The possible reasons for this are the increased discovery of low-dose radiation effects and the increased questioning of the applicability of the LNT model. After decades of research, the study of bystander effect-related phenomena has yielded breakthroughs and changes in paradigms, molecular mechanisms, and signaling events, ultimately leading to the development of the modern bystander effect theory (**Table 1**).

**Table 1:** The history of the study of radiation-induced bystander effect.

Time	Person	Events	References
1905	Heineke et al.	“Lymphoid Elements Stimulation” Hypothesis	/
1915	Murphy, Morton	Indirect Tumor Effects	(21)
1928	Muller	“X-Rays and Mutations”	(20)
1936- 1940	Timofeeff-Ressovsky, Zimmer, Delbruck	“Target Theory”, “The Green Pamphlet”	(22–25)
1954	Parsons et al.	Distant Effects	(26)
1957	Franklyn, Watson, Crick	“The Central Dogma”	/
1967	Hollowell, Littlefield	DNA as a Target for RIBE	(27, 28)
1986	Seymour et al.	Lethal Mutations in Distant Progeny	(29)
1992	Nagasawa, Little	Genomic Instability in Distant Progeny “Modern RIBE Studies”	(18)
1997	Mothersill, Seymour	Soluble Factors	(30)
1998	Azzam et al.	GJIC	(31)
2015	Le et al.	Biophoton Signalling	(32, 33)

Besides the RIBE studies conducted with low-LET (linear energy transfer) radiations, such as X-rays, β-rays, γ-rays, etc., many studies were also carried out with high-LET radiations such as alpha particles, neutrons, and heavy ions in the context of heavy ion radiotherapy and manned spaceflight, which are of significance for the improvement of both radiotherapy efficacy and radioprotection. As mentioned above, Nagasawa and Little used alpha particles to irradiate cells and found the RIBE for the first time (18), thus inspiring later researchers to study the modern “bystander effect”. Later in this review, we will discuss a number of experiments using low or high-LET radiations, and most of them have led to some important conclusions. For example, in experiments exploring markers of bystander effects, researchers have found that high-LET particles upregulate MAPKs in bystander cells more significantly compared to low-LET radiations (34). However, low- and high-LET radiations often co-exist in nature, forming a complex radiation environment that affects cells and organisms at the same time. Radiation workers such as astronauts are often exposed to a complex environment composed of gamma, neutron, proton and heavy ion radiation at the same time. Many previous studies have shown that low- and high-LET radiations can interact with each other and lead to results beyond expectations (35–38). So if we want to draw more accurate conclusions about the bystander effect, we have to consider the combined effects of low- and high-LET radiations, which are currently missing in most of the studies.

**Figure 1:**
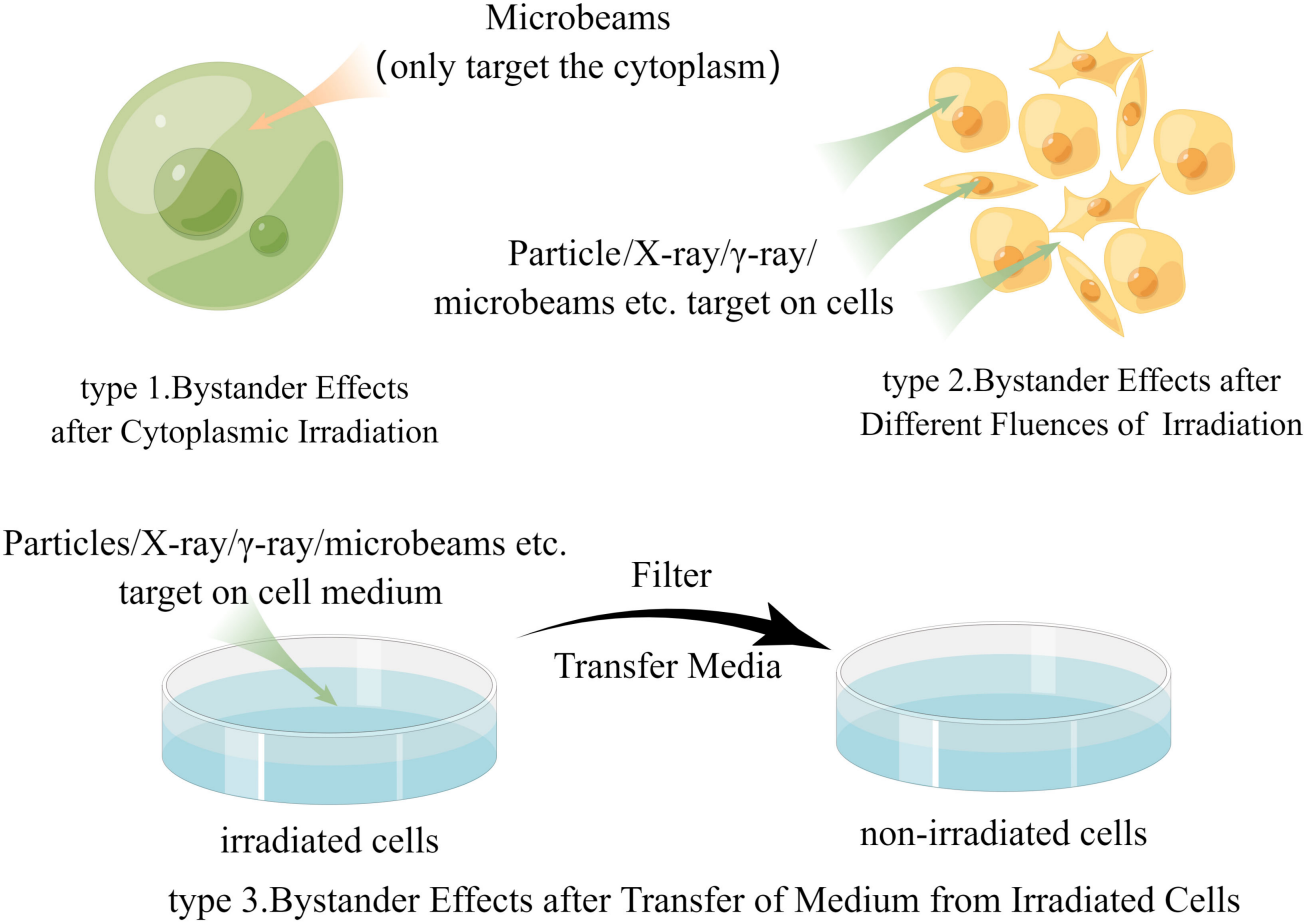
The main research methods of radiation-induced bystander effect (by Figdraw).

In the past few years, although some studies have obtained further direct evidence of bystander effects in animal experiments (39–41), the evidence from human samples is still inadequate. This may be because there are too many parameters in the field of radiation protection that dictate how much radiation is exposed to humans, including exposure dose, tissue type, radiation quality, and dose distribution. We question whether changing these factors can alter the likelihood of inducing bystander effects in humans; however, even after inducing bystander effects in humans, the weight of the effects of these factors is not yet known. Therefore, due to the lack of specific in-human bystander effect research results, most researchers mainly discuss data from *in vitro* experiments related to human exposure scenarios. These studies may be roughly categorized into three groups (**Figure 1**), and our discussion is mostly based on these experiments while some important animal experiments will also be discussed later.

## 3 Mechanisms of the bystander effect

A charged particle microbeam facility (42, 43) at the Gray Cancer Institute in the United Kingdom can enable the precise targeting of a single cell with a controlled dose of radiation, thereby realizing the measurement of radiation damage to a single cell. The laboratory measured the killing effect and the bystander effect manifested as micronucleus (MN) formation after irradiating primary human fibroblasts with charged particle microbeams and concluded that the indirect damage effect was even greater than the direct damage from radiation (44, 45). If the cells were not in direct contact, a bystander effect would also be found at a distance (44, 45). This shows that at least two distinct pathways are involved in RIBEs. Based on mechanistic studies of the bystander effects, the underlying mechanisms can be classified into two categories: (i) the existence of physical contact between irradiated and non-irradiated cells, which generates intercellular communication mainly through gap junctions (31), and (ii) the release of a series of soluble signaling molecules by irradiated cells that mediate the bystander effect in non-irradiated cells (30). Although the two have been divided into two research areas in most studies, at present they are not shown to be mutually exclusive. In fact, the two categories involve some common response steps that play a joint role in eliciting the bystander effect. However, a portion of the studies also suggests that physical signals may also be an important mechanism in the induction of the bystander effect. It has been found that UV photons from irradiated cells can also induce bystander effects in unirradiated cells (32, 33, 46–48). In terms of whether DNA is directly altered, most evidence suggests that epigenetic mechanisms play an important role in the bystander effect. Many studies have demonstrated the importance of epigenetic mechanisms in radiation-induced and maintained bystander effects from different perspectives by observing alterations in DNA methylation, histone methylation, and miRNA expression. In this section, we will discuss the results of *in vitro* and *in vivo* studies to explain the two main mechanisms involved in the bystander effect described above and their potential relationship with epigenetic alterations.

### 3.1 Gap junction-mediated bystander effect

As one of the most versatile cellular junctions, gap junctions are widely present among a variety of cells, and they allow cells to exchange information as well as small molecules. As a specialized membrane structure, gap junctions are low-resistance channels that connect adjacent cells, transmit electrical impulses in an excited state, and transport small molecules involved in metabolism and growth in a normal state (49, 50). The effect of gap junctional intercellular communication (GJIC) in bystander cells was first discovered by Azzam et al. in 1998 (31) and later confirmed experimentally in 2001 (51). The researchers exposed cells to low-flux alpha particles and observed the difference in bystander effects induced by the GJIC-blocked and normal groups, finally obtaining direct evidence for the involvement of GJIC in bystander effects.

At present, most teams investigate the relationship between GJIC and bystander effects from two perspectives: (i) the use of gap junction inhibitors, such as lindane, octanol, and dieldrin; and (ii) the use of cells deprived of gap junctions after gene editing to eliminate interference from GJIC. In experiments using gap junction inhibitors (52), human-hamster hybrid (A_L_) cells were exposed to a non-cytotoxic, mutagen-free dose of octanol (1 mM) 2 hours before and 3 hours after exposure to alpha particles. It was discovered that octanol decreased the induced CD59-mutant yield from 92 ± 35 to 16 ± 3 per 10^5^ survivors, indicating that the bystander effect was suppressed. Similarly, in another set of experiments, A_L_ cells and Chinese hamster ovary (CHO) cells were treated with a 40 μM dose of lindane, and it was also found that treatment significantly reduced the bystander effect (53, 54). Taken collectively, these findings indicate that GJIC appears to mediate the commencement of the bystander effect, but lindane and octanol are both non-specific inhibitors of the gap junction and may have an impact on other cellular processes (e.g., membrane function, etc.). Considering this, the researchers also used cells defective in connexin 43 for their experiments (connexin 43 is one of the proteins required for gap junction formation (55)). The bystander effect was eliminated or attenuated in cells with dominant negative connexin 43 (52).

The significance of the gap junction in the bystander effect has been questioned by several investigators in recent years. For example, Imaizum et al. observed in human lung cancer cell lines and rat cancer cell lines that the bystander effect was not altered by gap junction inhibitors or enhancers (56). Banaz-Yasar et al. found that the bystander effect in non-communicating Jeg3 malignant trophoblast cells was not affected by either gap junctions or connexin isoforms; Yang et al. demonstrated that the bystander effect induced in human fibroblasts was also not affected by gap junctions, as the cells were separated from each other (57).

The above experiments raise some questions about the role of GJIC in the bystander effect, and the results seem somewhat contradictory, but the fact remains that a different mechanism for the bystander effect may manifest in cells at different distances and in different locations. However, the results of these experiments also suggest that another mechanism may mediate the bystander effect: soluble signaling. We will go into more detail on the bystander impact caused by soluble signaling in the following sections.

### 3.2 Soluble signaling molecule-mediated bystander effects

#### 3.2.1 What are they?

Several studies have shown that soluble signaling molecules, in addition to gap junctions, are crucial in the transmission of bystander effects. The signaling process can be subdivided into the intercellular transmission of signals generated by irradiated cells and the intracellular signal transduction in bystander cells after receiving the signals.

#### 3.2.2 Intercellular signals

##### 3.2.2.1 Reactive oxygen species (ROS) and NAD(P)H

It is well known that some cellular organelles, such as mitochondria, endoplasmic reticulum, and peroxisomes, are involved in regulating the metabolic reactions of reactive oxygen species (ROS) (58–60). Normal cellular life activities depend on the balance of oxidants and antioxidants, and if this balance is disrupted, a state of oxidative stress occurs, leading to the development of a range of pathological phenomena, including cancer and degenerative diseases (61).

Nagasawa and Little (18) hypothesized that ROS were involved in the induction of sister chromatid exchange (SCE) and that this result could also be inhibited by superoxide dismutase (SOD), which inhibited the activity of SCE-promoting related cytokines in bystander cells, thus weakening the bystander effect. Later, a laboratory demonstrated by a more direct approach (hydroethidine probe to detect superoxide anion and 2’, 7’-dichlorofluorescein diacetate to detect hydrogen peroxide) that exposure of human cell cultures to low doses of alpha particles induced intracellular hydrogen peroxide as well as superoxide anion production *via* NAD(P)H oxidase (62). Studies using microbeams further supported the role of oxidative stress in the induction of DNA damage in α-particle irradiation-induced non-targeted effect (63). It was discovered that α-particle irradiation dramatically increased the number of CD59 point mutations in A_L_-CHO hybrid cells, whereas dimethyl sulfoxide (DMSO), a substance that scavenges free radicals, inhibited the mutations. The use of glutathione also reduced mutation induction (which acts as a redox buffer to prevent oxidative stress (64)).

Further data suggest that the upregulation of oxidative metabolism levels mediates the bystander effect in human fibroblasts. Using immunoblotting and *in situ* immunofluorescence to detect cellular p21^Waf1^ expression, Azzam et al. (65) found that active Cu-Zn SOD and active catalase inhibited the upregulation of p21^Waf1^ in human fibroblasts and micronuclei formation in bystander cells. The study also showed that metabolic ROS induced by alpha particles is involved in the signaling pathway that activates bystander effects. These experiments directly demonstrate how ROS and NAD(P)H contribute to the bystander effect.

##### 3.2.2.2 Interleukin (IL)-8

Interleukin (IL)-8, a cytokine of the chemokine family, is extensively involved in the life activities of organisms by binding to its specific receptor. In the experiment mentioned above (62), IL-8 was found to be associated with an increase in ROS levels. In another study by the same team, it was shown that exposure to a low dose of alpha particles (3.6–19 cGy) led to a significant increase in IL-8 protein production. Enzyme-linked immunosorbent assay (ELISA) and northern blot analysis showed that the increase of IL-8 protein after irradiation of normal human lung fibroblasts using alpha particles coincided with an increase in ROS levels. So the team hypothesized that the alpha particle-induced increase in IL-8 promoted an inflammatory response in the respiratory tract and was a key factor in promoting an increase in ROS levels.

By transferring the medium of irradiated cells to bystander cells, Facoetti et al. evaluated the role of IL-8 and its receptor (CXCR1) in the bystander effect of T98G cells after gamma irradiation, demonstrating that radiation could change IL-8 and CXCR1 expression levels in a non-dose but time-dependent manner (66). Subsequently, further experiments also demonstrated that the clonal survival of AG01522 and TG98 cells was significantly reduced at 5 h and 20 h after exposure to low doses of gamma radiation. Although there was no significant change in the concentration of IL-6, the amount of IL-8 released by the glioblastoma cells was significantly dependent on the amount of transfer medium, suggesting that IL-8 could influence the bystander effect (67).

##### 3.2.2.3 Transforming growth factor-beta1 (TGF-β1)

Iyer et al. mentioned that TGF-β1 and tumor necrosis factor alpha (TNF-α) were also involved in bystander signaling (68). Previous studies have shown that cellular oxidative stress caused by alpha particle irradiation can cause a rapid increase in the effective utilization of TGF-β1. Other studies have shown that TGF-β1 levels increase in low-dose radiation and other oxidative environments (69–72), and that substances may be released either by the rapid secretion of TGF-β1 or from silent-glycan receptors or an extracellular matrix (72–74). In terms of the long-term responses to radiation exposure, TGF-β1 is associated with the regulation of a range of inflammatory responses, which can ultimately influence the severity of inflammation in the body (75).

From a macromolecular perspective, TGF-β1 can associate with a bunch of clusterin (CLU) proteins, thereby mediating RIBEs (76). There are two main types of CLU, a secretory glycoprotein (sCLU) and a nuclear protein (nCLU). When nCLU is activated by TGF-β1 signaling, which involves the AP-1 transcription factor, there is a greater likelihood of apoptosis or quiescence (77, 78). On the other hand, however, TGF-β1 can also cause an increase in sCLU expression, which can act as a protective factor to block the TGF-β1-mediated bystander effect and thus perform a protective function (79). This suggests that CLU can play an opposing role in the bystander effect. These findings reveal the potential for TGF-β1 to crosstalk with other regulatory pathways to mediate the bystander effect

In recent years, studies have linked TGF-β1 to various small molecules to explore the oncogenic mechanisms of these molecules in bystander effects. Hu et al. (80) found that miR-663 was down-regulated in direct irradiation, but interestingly, in bystander cells, miR-663 was extensively upregulated. According to bioinformatic analysis, TGF-β1 is a direct target of miR-663. The researchers also found that miR-663 could directly target TGF-β1 to inhibit its expression, thereby suppressing the bystander effects. Besides, TGF-β1 was also associated with various lncRNAs (81). They used human lung bronchial epithelial cells (BEAS-2B) to demonstrate the important role of TGF-β1 in radiation-induced tumorigenesis and found that the crucial roles of TGF-β1 in the oncogenic transformation and tumorigenesis were regulated epigenetically by several lncRNAs. Taken together, these findings reveal the potential for TGF-β1 to crosstalk with other regulatory pathways to mediate the bystander effect. If future studies can map in detail the biological network of TGF-β1 in the mediation of the bystander effect, then TGF-β1 is likely to have important implications and breakthroughs for clinical radiation therapy.

##### 3.2.2.4 Nitric oxide (NO)

NO, which is mainly produced by different isoforms of nitric oxide synthase (NOS) with arginine as a substrate, is closely associated with vascular endothelial cells and nerve cells, and can cause muscle diastole by increasing cGMP and decreasing Ca^2+^ concentrations in smooth muscles through a series of processes. Because of its relative stability and hydrophobicity, NO is distinguished from other signaling molecules because it can diffuse among a few cells and does not need to cross intercellular connections to reach bystander cells. Many studies have shown that low dose radiation stimulates the production of NO and NOS. For instance, Matsumoto et al. found that the expression of inducible NOS (iNOS) was activated as early as 3 h after X-ray irradiation and continued to increase for 24 h after irradiation (82).

There are different isoforms of NOS, and Leach et al. found that activation of a combinatorial nitric oxide synthase (cNOS) induced early events in the bystander effect. After exposing CHO cells to 2-Gy X-ray irradiation, they found that the activity of cNOS was enhanced within just 5 minutes and returned to normal levels only after 30 minutes (83). In addition, MN formation has been demonstrated as an important event in the bystander effect, and Shao et al. observed a significant increase in MN formation in bystander cells after irradiating human salivary gland tumor cells (HSG) with alpha particles. To verify the role of NO, they used the NO scavenger 2-(4-carboxyphenyl)-4,4,5,5-tetramethylimidazoline-1oxyl-3-oxide (c-PTIO) to eliminate excess MN production after pretreatment (84, 85), clearly suggesting that NO has a key role in MN formation. Other studies (86) have also shown that MN as well as double-strand breaks (DSBs) increased in bystander cells upon irradiation with low doses of alpha particles (10 cGy), where NO played an important role.

#### 3.2.3 Intracellular signaling pathways

##### 3.2.3.1 COX-2 related cascade reactions

Before discussing the COX-2-related cascade response, it is important to demonstrate the role of COX-2 in the bystander effect. Zhou et al. (87) used normal human lung fibroblasts (NHLF) and NS-398, a specific COX-2 inhibitor, to examine bystander effects and found that there were 4.2 ± 1.2 bystander effect-induced mutations per 10^6^ surviving cells in the control group, whereas bystander effect-induced mutations were reduced by more than 6-fold to 0.7 ± 0.2 per 10^6^ surviving cells in the NS-398-treated group (87). The activation of the mitogen-activated protein kinase (MAPK) signaling cascade by insulin growth factor and other cytokines, and the phosphorylation of extracellular signal-related kinase (ERK) were found to be upstream events for the increase in COX-2 expression. The researchers discovered that at 4 hours after irradiation, phosphorylated ERK levels were significantly upregulated in both normal and bystander NHLF cells. In addition, the ratio of phosphorylated ERK to normal ERK increased from 2 to 13 in bystander cells, whereas MAPK p38 kinase activity increased at 4 hours and remained elevated at 16 hours. If these two kinases are indeed upstream molecules in the pathway of the bystander effect, then they can be inhibited by relevant inhibitors. For example, treatment with a specific MAPK kinase (MEK)-ERK inhibitor, PD 98059, could almost completely inhibit the bystander effect.

In summary, the binding of TNF-α, IGF, and various leukotrienes to cell surface receptors activates the MAPK signaling pathway, which in turn upregulates COX-2 downstream, thereby mediating the bystander effect. Several studies have also shown that MAPK and NF-κB transcription factors alone or together can stimulate the expression of COX2 and NOS in the nucleus (68, 88, 89).

##### 3.2.3.2 Intracellular calcium fluxes

The activation of the calcium influx pathway increases the intracellular calcium flux in cells. Lyng et al. (88) used two fluorescent calcium-sensitive molecular probes, Fluo 3 and Fura Red (Fluo 3 binds calcium with enhanced green fluorescence and Fura Red binds calcium with reduced red fluorescence (90)), to measure calcium levels. They incubated the cells with Fluo 3 and Fura Red for 1 hour and found that the irradiated cell conditioned medium (ICCM) increased the celluar calcium level. The calcium level could be inferred by measuring the ratio of Fluo 3 to Fura Red. The basal calcium ion concentration was 106.2 ± 2.2 nM (n=12). When calcium ionophore A23187 was added to the induced cells, the calcium level increased from 283.8 ± 5.7 to 342.3 ± 9.7 nM compared to the 0.5 Gy ICCM group (88).

To verify the role played by calcium influx in the death of bystander cells, the investigators inhibited calcium inward flow with the calcium channel blockers EGTA and phenylalkylamine (verapamil), thus observing different phenomena. Significant apoptosis was observed in ICCM-induced HPV-G cells, whereas cells induced by ICCM after the addition of EGTA or phenylalkylamine did not produce significant apoptosis (88).

Shao et al. also treated T98G and AG0 cells with the highly calcium-selective A23187 vector and found that the intracellular calcium concentration increased to 140 ± 15 nM in T98G cells and 186 ± 10 nM in AG0 cells, and rapid calcium fluxes within 30 seconds were partially observed in both cell types. However, when the team used ICCM to induce the bystander effect, they found that T98G cells could not increase calcium flux when NOS was inhibited by the non-selective inhibitor aminoguanidine, suggesting that NO may be involved in the induction of calcium influx in response to conditioned medium in T98G cells.

Moreover, cells exposed to ICCM and pretreated with inhibitors of calmodulin or inhibitors of mitochondrial calcium uptake can inhibit the production of γ-H2AX and NO in the bystander effect (91). These results suggest that calcium inward flow might regulate the downstream events of γ-H2AX and NO and finally mediate the bystander effect.

##### 3.2.3.3 Nuclear factor-kappa B activation (NF-κB) activation

Ataxia telangiectasia mutated (ATM) kinase, an autophos-phorylated protein that is normally found as an inactive dimer in the nucleus and cytoplasm, functions in DSB repair. After the radiation-induced production of DSBs in genomic DNA, downstream ATM-related events are initiated (92–94). ATM directly phosphorylates three related proteins, p53, CHK2, and MDM2, which are involved in the regulation of p53 function and levels and are among the key events affecting irradiated cells. The RIBE of ATM/ATR (ATM and Rad3-related) has been described (95). In addition, ATM initiates downstream events that cause cell cycle arrest and apoptosis by affecting mitochondria-related functions (96).

However, NF-κB proteins, first discovered by David Baltimore (97–99), are a family of proteins that selectively bind to the κ-light chain enhancer of B cells to regulate the expression of many genes. Upon initiation in the nucleus, NF-κB can activate the ATM kinase-mediated pathway (100), which can rapidly upregulate NF-κB-dependent gene expression (100–102), including IL-6, IL-8, and TNF-α.

In another study, the TNF/TNF-R1 pathway was found to activate the inhibitor κB kinase (IKK) complex *via* the TRADD/TRAF2/RIP complex-1, thereby targeting NF-κB (IκB) inhibitors and regulating gene expression after delivering NF-κB into the nucleus (103–105). In addition, NF-κB-dependent IL-6 expression can also establish a link between ATM, NF-κB, and signal transducer and activator of transcription 3 (STAT3) in another pathway (103, 104).

## 4 Several important issues in the study of RIBE

Up to now, a number of studies have attempted to demonstrate the mechanisms underlying RIBE and some specific biological molecules involved have been identified, however, there are still some issues in RIBE to be resolved, which are important to both a better understanding of RIBE and the practical modulation of RIBE in the fields of radiotherapy and radioprotection. We have focused on these issues and discussed them below in the hope of providing some ideas and directions for future researchers.

### 4.1 Triggering of bystander signals

Many studies now use various doses for parallel comparisons rather than attempting to understand bystander signals at a single dose as they did in earlier research. Some have produced interesting results, but the relationship between the magnitude of the induced effect and the dose cannot yet be determined accurately.

Giuseppe et al. (106) irradiated individual cells with X-rays of less than 0.5 Gy and found that the likelihood of inducing bystander effects increased with increasing radiation dose, whereas once a response was induced, their effects were nearly equal. Interestingly, however, if the dose was less than 0.3 Gy, the dose-effect curve changed considerably, with the likelihood of triggering a bystander effect decreasing with increasing irradiation dose and showing an “all or none” response. For alpha particles, a single particle passing through a cell is adequate to induce the bystander effect (45). When cells were exposed to single cell microbeam, Sawant et al. discovered (107) that the frequency of morphological transformations was roughly comparable irrespective of whether 10% or 100% of the cells were transversed. Additionally, several studies have shown that the bystander effect induced by exposing cells to either one or many alpha particles is similar (44, 85, 108, 109), a finding that is consistent with other heavy ion irradiations (110–112). For beta particles, a dose-dependent bystander effect was also observed when their radionuclide activity was increased (113). In medium transfer experiments (114), human keratin-forming cells were irradiated with 0.01–0.5 Gy low-LET cobalt 60 radiation followed by medium transfer, after which the clonal survival and number of cells killed after medium transfer were measured. It was found that the bystander effect appeared saturated in the range of 0.03–0.05 Gy. By contrast, once the irradiation dose was greater than 0.5 Gy, the resulting death curve was due to a combination of the direct effect of irradiation and a non-dose-dependent bystander effect.

Taken together, these findings indicate that bystander effects involve an “all or none” switch mechanism (106, 115–117). Once triggered, it does not depend on the irradiation dose and exhibits approximately the same response effect, which can last for a long time and be transmitted to the next generation as the cells reproduce (118–120). Even more intriguingly, the created signal can elicit reactions in various species (121, 122), indicating that the response is highly conserved during evolution and may be one of the most basic ways that individuals react to their surroundings.

One explanation for the saturation of the bystander effect is the limitation of the signaling molecules that can be produced by the irradiated cells. As long as the dose threshold is reached, even if the irradiation dose is further increased, no other signaling molecules can be induced. If the dose threshold is reached at the beginning of the specified irradiation, then the subsequent responses are dose-independent (within a certain range). Another explanation is that although there is a positive relationship between the induction of the effect and the dose within a certain range, the bystander cells are already completely responsive to the signaling molecules produced by the induction and are no longer sensitive to any subsequent signals.

Although the “all or none” nature of the bystander effect has been extensively studied, it is still not precisely defined because there may be a series of chain reactions in the signaling cascade, and each of these reactions may have a corresponding threshold, which eventually leads to the macroscopic “all or none” nature of the bystander effect.

### 4.2 How long can the signaling molecule exist? What are the exact dynamics of the changes?

Findings suggest that bystander signals can remain active in organisms for extended periods of time. For instance, in the plasma of atomic bomb survivors, 10.9% of leukocyte chromosomes were found to be broken, which was significantly more than that in control individuals (123). The study showed that the factors related to bystander effects remained active for at least 30 years after radiation exposure. In the Chernobyl accident, factors detected in the blood of survivors that could cause chromosomal breaks persisted for at least 9–20 years (124, 125). Researchers have endeavored to isolate and explore the nature of these factors, but convincing results have yet to be reported.

Experimental methods that have been used for bystander effect characterization include cell clone formation assays, SCE assays (18), and locus mutation assays (126). The average time it takes to reach the experimental endpoint varies, but, in general, it takes anywhere from a few minutes to several days or even weeks. As a result, it has been challenging to determine the precise values of time for the development, existence, and reception of bystander effect signals. Moreover, the measurement of certain signals is still not known. For longer bystander signals, it is likely that other biological signals are interfering with the response, making it much more difficult to identify the precise bystander signal. Most experiments currently focus on measuring the peak concentrations of the bystander signal, which are more sensitive and easier to test, or analyzing changes in cell phenotype (e.g., colony aggregation, clonal survival) to infer the endpoint of the bystander signal-induced response, although few experiments can specifically account for the dynamic nature of the signal.

Fortunately, as research on RIBE has progressed, a framework for assessing RIBE within a few hours has now been established. This enables researchers to detect the rapid production (within 5 minutes) of DSBs (127–129) or the expression of specific genes or associated proteins in bystander cells to assess the degree of risk. One thing that can be ascertained is that the increased knowledge of the specific timing of bystander signaling, transmission, and reception can broaden our overall knowledge of bystander effects. The following sections discuss some findings on the timing of bystander signaling in general terms.

The family of histones includes the H2AX proteins. One of the early responses of cells to DSBs caused by various injuries is the phosphorylation of γ-H2AX. Sedelnikova et al. assessed H2AX levels in whole cells by counting the number of γ-H2AX in the nucleus (130). This approach is currently one of the most sensitive methods to assess DSBs, and it has been used by several investigators to examine RIBE (57, 131–134). For example, within 1 hour of iron ion irradiation, Yang et al. observed the formation of γ-H2AX foci in bystander cells in a co-cultured system that last for more than 24 hours (135). It was also found that γ-H2AX foci were significantly higher in non-irradiated bystander cells within 10 min than in controls and peaked after 30 min (127, 129), suggesting that γ-H2AX could act as a rapid signal to induce RIBE. In other respects, the connection between the formation of γ-H2AX foci and DSB aids in our evaluation of RIBE injury.

Some researchers have observed that 53BP1 can co-localize with γ-H2AX in bystander human skin fibroblasts (136). Therefore, using this connection, many teams have assessed RIBE with 53BP1 *in vitro* and *in vivo*. For example, Tartier et al. found that the proportion of 53BP1-positive bystander cells peaked 1–3 h after the onset of irradiation.

Phosphorylated ATM can also co-localize with γ-H2AX. RIBE is eliminated entirely if ATM is not functional (95, 136). In another study, ATM foci in bystander cells can be identified as early as 0.05 hours after X-ray exposure and last for at least 48 hours at levels that are much higher than in controls (4–5-fold higher). In line with expectations, DSB repair induced by direct irradiation was faster than that induced by bystander effects (137). Most studies examining the generation, transmission, and decay of bystander signals often use ICCM, as it allows for the rapid detection and collection of relevant signal parameters. In terms of the critical time point for bystander signaling, some current opinions suggest that the transduction of the signal is somewhat rapid. Within 30–60 seconds of irradiation, irradiated cells or irradiated medium have been separated from non-irradiated cells, and it has been discovered that the likelihood of bystander effects manifesting was greatly decreased (108, 138).

However, although different studies have given a certain “time” for the bystander effect, measurements of different signaling substances can still result in large differences, and in the absence of information on these signaling molecules, it is not possible to determine whether the bystander effect in various cells is caused by a single signal or multiple signals acting together. It is also not possible to determine whether bystander signals are caused by the accumulation of low doses of radiation over long periods of time. These unanswered questions provide directions for future research in the field, whether it is possible to conduct experimental studies at different time intervals and upon different numbers of exposures to simulate the dynamics of bystander signaling more realistically.

### 4.3 Is the induction of bystander signaling radiation source-dependent or cell-dependent?

The sources of radiation used in the different studies investigating bystander effects vary, as do the doses used by each source. The most typical sources of radiation are alpha particles, beta particles, X-rays, and carbon ion radiation, as demonstrated in the experimental studies mentioned above. In addition, some studies have also used neutrons and other sources of elemental radiation to observe bystander effects. Given that most studies generally use the same radiation source to ensure more predictable results, this makes it more difficult to compare the relationships between individual radiation sources horizontally.

In terms of recent experimental data, changing the type of radiation source within the same study induces different bystander effects. Generally speaking, changing the intensity of a single radiation source causes a change in the magnitude of the bystander effect (84, 139, 140). However, no bystander effect is induced on cells even when the type of radiation source is changed (141, 142). Thus, it is possible that the selection of bystander effect markers in these studies heavily relied on DNA damage indicators. Although the indicators of DNA damage, such as MN formation and γ-H2AX foci, are crucial for bystander effects, it is possible that the effects induced in this experiment were not strong enough to be detected by these means. However, the nature of the phenomenon will be further elucidated if future studies can be conducted with a variety of radiation sources to explore contradictory occurrences.

With regard to the induction of bystander signals, there are also varying degrees of interaction between the radiation source and cell type. The initial studies included different cell types from species such as hamsters, humans, mice, and rats. In current studies, the cell types that are used differ depending on the purpose of the study, and many normal primary cells, transformed cells, and tumor cell lines have been investigated. However, the greatest difficulty in elucidating the differences in RIBEs for different cell lines, as with verifying the source dependence of radiation in bystander effects, is the diverse settings used for different experiments.

In summary, these studies show that the radiation sources or cell types have a complex relationship with the bystander effect. Unless the definition and endpoints of bystander effects are finally harmonized, it will be difficult to obtain reliable results on the dependence of radiation sources and cell types on bystander effects.

## 5 Bystander effects *in vivo*

Although the results of *in vitro* experiments are mostly different from those of *in vivo* experiments, it is only when the mechanisms of *in vivo* experiments are validated that bystander effects can be applied to the assessment and treatment of radiation protection in practice. The several possible mechanisms of the bystander effect have been discussed in detail above. The mechanisms of *in vitro* experiments can mostly explain the results of *in vivo* experiments, and animal studies mostly support the role of epigenetic mechanisms in the induction and maintenance of the bystander effect.

Koturbash et al. (143) placed one side of a mouse body in a medical protection device while the other side was directly exposed to radiation to observe changes in DNA methylation and protein expression in the exposed and non-exposed skin. Direct exposure suppressed the total methylation level in the irradiated tissue, whereas the changes in bystander skin tissue were not significant. Interestingly, there was a significant decrease in *de novo* DNA methyltransferases (DNMTs) detected in bystander skin but a significant increase in two methyl-binding proteins, MeCP2 and MBD2, which are associated with transcriptional silencing. To further investigate the epigenetic alterations in animals by local exposure, the investigators exposed rats to 20 Gy localized cranial irradiation in another experiment (144) and observed the induction of epigenetic changes at 24 hours as well as 7 months later. They found that confined cranial radiation caused severe epigenetic dysregulation in splenic tissues distantly under radiation shielding, including an overall decrease in DNA methylation and the overall down-regulation of DNA transferases, and the hypomethylation effect could persist for up to 7 months. It was also found that intracranial irradiation of mice also caused significant changes in p53 aggregation and apoptosis levels in the spleens (145).

More interestingly, recent studies have found that bystander effect signaling may be sex differential in animals (146). In a study of female/male mice exposed locally and systemically, researchers found that specific microRNAs were expressed in the spleen of female/male mice and that the microRNAs were sex differential, suggesting that sex hormones may play an important role in the RIBE.

## 6 Clinical implications of bystander effect studies

With the development of medical technologies, the theories and types of radiotherapy are gradually diversifying. Radiotherapy is not limited to the use of conventional photons such as X-rays but is gradually developing the practice of particles such as protons and carbon ions. High-energy protons, as well as heavier particles, such as carbon ions, have a more satisfactory energy deposition and greater biological effect than conventional photon therapy. For photon radiotherapy, the peak dose is deposited in the normal tissue and then the dose gradually decreases, meaning that the normal tissue is exposed to a certain dose throughout the irradiation path. In contrast, proton and carbon ion therapy tend to allow the dose to be deposited mainly in the lesion due to the Bragg peak, resulting in a substantially lower dose deposition in normal tissue than photon therapy. The lower dose deposition to non-targeted tissues not only results in a subsequent reduction in radiation-induced toxic effects, but also reduces the incidence of bystander effects in non-irradiated areas. These advantages will improve patients’ tolerance to radiotherapy and thus improve patient outcomes. It has been shown that in younger patients, proton therapy can reduce the deposition dose to normal tissues by approximately 60%, which can reduce the incidence of secondary malignancy (147). A team of researchers used X-rays as well as carbon, neon, and argon ions to investigate bystander effects and found that heavy ions increased the frequency of micronuclei in bystander cells compared to X-rays, thus triggering concern about increased genomic instability and secondary carcinogenesis probability (148). However, more comprehensive *in vitro* and *in vivo* data comparing RIBE induced by different radiations are needed to improve radiotherapy outcomes and relevant radioprotection.

During the practical radiotherapy of cancer, it is important to protect the normal tissue within the non-irradiated area, thereby reducing the bystander effect. However, in the process of protection, it is also crucial that this protection should not affect the ability of radiation to kill tumor cells. An up-and-coming targeted therapy in the field is the use of gene therapy to deliver radionuclides directly to tumor cells (149–151), intended to apply a signal amplification effect to increase the killing capacity of the rays. However, this therapy still requires the certainty that the induced response is apoptotic rather than protective and ultimately has a positive effect on the therapy.

To overcome the limitations of *in vitro* experiments and demonstrate whether radionuclide-induced bystander effects can occur directly *in vivo*, Xue et al. (152) used 5-[(^125^)I]iodo-2’-deoxyuridine (^125^IUdR) to label tumor cells. Because DNA-bound ^125^IUdR has a certain attenuation range (<0.5 μm), which causes little direct radiation to unirradiated cells, the alterations in nearby cells were considered to be caused by bystander effects. Meanwhile, a mixture of human colon LS174T adenocarcinoma cells and LS174T cells labeled with a lethal dose of ^125^IUdR DNA was injected into nude mice, and a significant inhibitory effect on subcutaneous tumor cells was found. However, the experiment is noteworthy in that the use of lethal doses of ^125^IUdR-labeled DNA at the beginning may directly cause cell death, thereby releasing radioactive material to have a direct irradiation effect on cells, which may cause some experimental errors.

Many radionuclides do not induce identical bystander effects *in vivo*, i.e., radionuclides induce bystander effects *in vivo* that are both tumor-killing and tumor-promoting. Kishikawa et al. (153) implanted nude mice with LS174T adenocarcinoma cells and reduced LS174T growth by DNA tagged with ^125^I cell growth (equivalent to the inhibition of the bystander effect). However, when the above experiments were repeated using ^123^I, it was found that the decay of ^123^I in tumor cells stimulated the proliferation of unlabeled tumor cells (equivalent to promoting the bystander effect). Both isotopes emit oxygen electrons, but the difference in their half-lives leads to different dose rates, with the latter being 109 times higher than the former. So that seemingly contradictory effects can be analyzed from a dosimetric point of view. If further conclusions are desired, then the bystander effects of different nuclides at different doses can be examined. Iodine is an element that is often used not only in animal experiments but also in clinical aspects where several of its isotopes play important roles. For example, ^131^I decay usually emits beta rays and can be used as a potential contrast and therapeutic agent for neuroblastoma (152, 154), whereas ^131^I-labeled NaI can be used to treat well-differentiated thyroid cancer (154).

Currently, therapeutic agents that are clinically available and use theories related to the bystander effect include antibody-drug conjugates (ADCs). Paul Ehrlich first presented the notion for this drug at the start of the twentieth century (155), and a modern version of ADCs has been made possible by advancements in bioengineering and related pharmaceutical procedures. However, the first treatment of solid tumors with ADCs occurred in 2013 with ado-trastuzumab emtansine (T-DM1), which targets the human epidermal growth factor 2 receptor (HER2) in metastatic breast cancer. To address the metastatic characteristics and diverse gene expression profiles of tumors, cleavable junctions and hydrophobic carriers have been developed through the ongoing enhancement of monoclonal antibodies and optimization of drug linkages, both of which mainly use the bystander effect to kill tumor cells.

## 7 Conclusion and future prospects

In summary, the effects of low doses of radiation on humans cannot be explained solely in terms of direct targeting of the cell nucleus. This is because the cells that were not directly exposed to radiation also presented high levels of genetic mutations, chromosomal variations, and cell lethality in their progeny, a phenomenon known as radiation-induced genetic instability (RIGI) (156). RIGI is a type of non-target effect that is similar to RIBE. Direct DNA damage is undeniably frequent in most tests, despite the majority of attention in this article being paid to documenting the phenomena of bystander effects in specific experiments. Genetic instability is thought to be a risk factor for the development of cancer, and it remains difficult for the body to process relevant signals in the cell to reduce the accumulation of DNA damage. The cell cycle checkpoint-related genes CCNB1 and RAD51 are overexpressed in bystander cells (31), and pathways such as ATR, ATM, and CHK1 are potential targets for regulating genotoxicity in bystander cells (95, 157, 158). In most cases, the cellular response to DNA damage is mediated by various protein kinases, including ATR and ATM. ATR primarily targets downstream CHK1 to prevent cells that have been genetically damaged (e.g. after radiotherapy) from entering mitosis. In detail, when DSB is generated, the MRE11/NBS1/RAD51 complex can promote S-phase cell cycle arrest and the p53-associated G1/S-phase checkpoint *via* activating the ATM/CHK2 pathway (159). However, the ATR/CHK1 pathway is activated when single-strand DNA damage occurs, triggering intra-S- and G2/M-phase checkpoints (160–162). Since most cancer cells have dysregulated G1 checkpoints, they are mostly dependent on S and G2 checkpoints activated by the ATR/CHK1 pathway.

Dahle et al. (163) noted regular increases in ROS in the offspring of radioactive cells, indicating that potentially a modified version of SOD-like principles could be used in humans to lessen radiation-caused injury to surrounding healthy tissues. Similarly, the use of inhibitors that target the above-mentioned molecules can lead to a reduction or elimination of the bystander effect (18, 64). In other words, to apply RIBE and other non-targeted effects in clinical treatment, the process of RIBE, RIGI, and abscopal effect should be artificially regulated. However, this necessitates an adequate recognization of the mechanism and signaling pathways involved in RIBE. Nowadays, some radioprotective agents have been developed to reduce damage from RIBE. For example, DNA-binding agents, like meprobamate, prevent DNA damage by acting as a reducing agent in electron transfer and have been proved by Burdak et al. that it can prevent RIBE (164).

In summary, using the bystander effect as an entry point can give us new insights into radiation therapy and the associated off-target effects. Exploring the key molecular pathways, as well as specific signaling molecules, would be extremely beneficial for the clinical sensitization to radiation therapy and radiation protection.

## Author contributions

Conceptualization, WH, HB, and HH; Literature Retrieval and Analysis, XH, ZN, and HTH; Writing—Original Draft Preparation, HT; Visualization, HT and LC; Writing—Review and Editing, WH, HB, and HH; Supervision, HH. All authors contributed to the article and approved the submitted version.

## References

[1] Ichikawa R. Current activities of united-nations scientific committee on the effects of atomic radiation. J Atomic Energy Soc Japan (1986) 28(2):134–8. doi: 10.3327/jaesj.28.134

[2] Spatola GJ Buckley RM Dillon M Dutrow EV Betz JA Pilot M. The dogs of Chernobyl: Demographic insights into populations inhabiting the nuclear exclusion zone. Sci Adv (2023) 9(9):eade2537. doi: 10.1126/sciadv.ade2537 36867701 PMC9984172

[3] Morgan WF Sowa MB. Non-targeted bystander effects induced by ionizing radiation. Mutat Research-Fundamental Mol Mech Mutagenesis (2007) 616(1-2):159–64. doi: 10.1016/j.mrfmmm.2006.11.009 17134726

[4] Bright S Kadhim M. The future impacts of non-targeted effects. Int J Radiat Biol (2018) 94(8):727–36. doi: 10.1080/09553002.2018.1454617 29569509

[5] Campa A Balduzzi M Dini V Esposito G Tabocchini MA. The complex interactions between radiation induced non-targeted effects and cancer. Cancer Lett (2015) 356(1):126–36. doi: 10.1016/j.canlet.2013.09.030 24139968

[6] Kaminski CY Dattoli M Kaminski JM. Replacing LNT: The integrated LNT-hormesis model. Dose-Response (2020) 18(2):1559325820913788. doi: 10.1177/1559325820913788 32313523 PMC7160778

[7] Ricci PF Tharmalingam S. Ionizing radiations epidemiology does not support the LNT model. Chemico-Biological Interact (2019) 301:128–40. doi: 10.1016/j.cbi.2018.11.014 30763555

[8] Calabrese EJ Hanekamp JC Shamoun DY. The EPA cancer risk assessment default model proposal: Moving away from the LNT. Dose-Response (2018) 16(3):1559325818789840. doi: 10.1177/1559325818789840 30116166 PMC6088500

[9] Shuryak I Sachs RK Brenner DJ. Quantitative modeling of carcinogenesis induced by single beams or mixtures of space radiations using targeted and non-targeted effects. Sci Rep (2021) 11(1):23467. doi: 10.1038/s41598-021-02883-y 34873209 PMC8648899

[10] Fakir H Hofmann W Tan WY Sachs RK. Triggering-response model for radiation-induced bystander effects. Radiat Res (2009) 171(3):320–31. doi: 10.1667/rr1293.1 19267559

[11] Richard M Webb RP Kirkby KJ Kirkby NF. A computer model of the bystander effect: effects of individual behaviours on the population response. Appl Radiat Isot. (2009) 67(3):440–2. doi: 10.1016/j.apradiso.2008.06.037 18845443

[12] Shuryak I Sachs RK Brenner DJ. Biophysical models of radiation bystander effects: 1. spatial effects in three-dimensional tissues. Radiat Res (2007) 168(6):741–9. doi: 10.1667/rr1117.1 18088188

[13] Schöllnberger H Mitchel RE Crawford-Brown DJ Hofmann W. A model for the induction of chromosome aberrations through direct and bystander mechanisms. Radiat Prot Dosimetry (2006) 122(1-4):275–81. doi: 10.1093/rpd/ncl433 PMC3088355 17166875

[14] Little MP Filipe JA Prise KM Folkard M Belyakov OV. A model for radiation-induced bystander effects, with allowance for spatial position and the effects of cell turnover. J Theor Biol (2005) 232(3):329–38. doi: 10.1016/j.jtbi.2004.08.016 15572058

[15] Olobatuyi O de Vries G Hillen T. A reaction-diffusion model for radiation-induced bystander effects. J Math Biol (2017) 75(2):341–72. doi: 10.1007/s00285-016-1090-5 28035423

[16] Khvostunov IK Nikjoo H. Computer modelling of radiation-induced bystander effect. J Radiol Prot (2002) 22(3a):A33–7. doi: 10.1088/0952-4746/22/3a/306 12400944

[17] Brenner DJ Little JB Sachs RK. The bystander effect in radiation oncogenesis: II. a quantitative model. Radiat Res (2001) 155(3):402–8. doi: 10.1667/0033-7587(2001)155[0402:tbeiro]2.0.co;2 11182790

[18] Nagasawa H Little JB. Induction of sister chromatid exchanges by extremely low doses of alpha-particles. Cancer Res (1992) 52(22):6394–6. 1423287

[19] Murphy JB Morton JJ. The effect of roentgen rays on the rate of growth of spontaneous tumors in mice. J Exp Med (1915) 22(6):800–3. doi: 10.1084/jem.22.6.800 PMC2125377 19867960

[20] Muller HJ. The production of mutations by x-rays. Proc Natl Acad Sci United States America (1928) 14:714–26. doi: 10.1073/pnas.14.9.714 PMC1085688 16587397

[21] Murphy JB Morton JJ. The effect of roentgen rays on the rate of growth of spontaneous tumors in mice. J Exp Med (1914) 22(6):800–3. doi: 10.1084/jem.22.6.800 PMC2125377 19867960

[22] Timofeeff-Ressovsky NW Zimmer KG. Neutron radiation trials for the triggering of mutation on drosophila melanogaster. Naturwissenschaften (1938) 26:362–5. doi: 10.1007/bf01774257

[23] Timofeeff-Ressovsky NW. Chemical-biological applications of flip neutrons and artificial radioactivity materials. Angewandte Chemie (1941) 54:437–42. doi: 10.1002/ange.19410544102

[24] Zimmer KG Timoeeff-Ressovsky NW. Note on the biological effects of densely ionizing radiation. Phys Rev (1939) 55(4):0411–1. doi: 10.1103/PhysRev.55.411

[25] Delbruck M. Radiation and the hereditary mechanism. Am Nat (1940) 74:350–62. doi: 10.1086/280901

[26] Parsons WB Watkins CH Pease GL Childs DS. Changes in sternal marrow following roentgen-ray therapy to the spleen in chronic granulocytic leukemia. Cancer (1954) 7(1):179–89. doi: 10.1002/1097-0142(195401)7:1<179::Aid-cncr2820070120>3.0.Co;2-a 13126913

[27] Hollowell JG Littlefield LG. Chromosome damage induced by plasma of X-rayed patients - an indirect effect of X-ray. Proc Soc Exp Biol Med (1968) 129(1):240–+. doi: 10.3181/00379727-129-33295 5686521

[28] Littlefield LG Hollowell JG Pool WH. Chromosomal aberrations induced by plasma from irradiated patients - an indirect effect of X radiation - further observations and studies of a control population. Radiology (1969) 93(4):879–+. doi: 10.1148/93.4.879 5824247

[29] Seymour CB Mothersill C Alper T. High yields of lethal mutations in somatic mammalian-cells that survive ionizing-radiation. Int J Radiat Biol (1986) 50(1):167–79. doi: 10.1080/09553008614550541 3487520

[30] Mothersill C Seymour C. Medium from irradiated human epithelial cells but not human fibroblasts reduces the clonogenic survival of unirradiated cells. Int J Radiat Biol (1997) 71(4):421–7. doi: 10.1080/095530097144030 9154145

[31] Azzam EI de Toledo SM Gooding T Little JB. Intercellular communication is involved in the bystander regulation of gene expression in human cells exposed to very low fluences of alpha particles. Radiat Res (1998) 150(5):497–504. doi: 10.2307/3579865 9806590

[32] Le M Mothersill CE Seymour CB Ahmad SB Armstrong A Rainbow AJ. Factors affecting ultraviolet-a photon emission from β-irradiated human keratinocyte cells. Phys Med Biol (2015) 60(16):6371–89. doi: 10.1088/0031-9155/60/16/6371 26237407

[33] Le M McNeill FE Seymour C Rainbow AJ Mothersill CE. An observed effect of ultraviolet radiation emitted from beta-irradiated HaCaT cells upon non-beta-irradiated bystander cells. Radiat Res (2015) 183(3):279–90. doi: 10.1667/rr13827.1 25710575

[34] Dong C He M Ren R Xie Y Yuan D Dang B. Role of the MAPK pathway in the observed bystander effect in lymphocytes co-cultured with macrophages irradiated with γ-rays or carbon ions. Life Sci (2015) 127:19–25. doi: 10.1016/j.lfs.2015.02.017 25748424

[35] McNally NJ de Ronde J Folkard M. Interaction between X-ray and alpha-particle damage in V79 cells. Int J Radiat Biol Relat Stud Phys Chem Med (1988) 53(6):917–20. doi: 10.1080/09553008814551281 3259561

[36] Mason AJ Giusti V Green S Munck af Rosenschöld P Beynon TD Hopewell JW. Interaction between the biological effects of high- and low-LET radiation dose components in a mixed field exposure. Int J Radiat Biol (2011) 87(12):1162–72. doi: 10.3109/09553002.2011.624154 21923301

[37] Staaf E Deperas-Kaminska M Brehwens K Haghdoost S Czub J Wojcik A. Complex aberrations in lymphocytes exposed to mixed beams of (241)Am alpha particles and X-rays. Mutat Res (2013) 756(1-2):95–100. doi: 10.1016/j.mrgentox.2013.05.001 23669292

[38] Staaf E Brehwens K Haghdoost S Nievaart S Pachnerova-Brabcova K Czub J. Micronuclei in human peripheral blood lymphocytes exposed to mixed beams of X-rays and alpha particles. Radiat Environ Biophys (2012) 51(3):283–93. doi: 10.1007/s00411-012-0417-x 22526916

[39] da Silva PFL Ogrodnik M Kucheryavenko O Glibert J Miwa S Cameron K. The bystander effect contributes to the accumulation of senescent cells *in vivo* . Aging Cell (2019) 18(1):e12848. doi: 10.1111/acel.12848 30462359 PMC6351849

[40] Olivares A Alcaraz-Saura M Achel DG Berná-Mestre JD Alcaraz M. Radiation-induced bystander effect: Loss of radioprotective capacity of rosmarinic acid *In vivo* and *in vitro* . Antioxidants (Basel) (2021) 10(2):231. doi: 10.3390/antiox10020231 33546480 PMC7913630

[41] Hargitai R Kis D Persa E Szatmári T Sáfrány G Lumniczky K. Oxidative stress and gene expression modifications mediated by extracellular vesicles: An *in vivo* study of the radiation-induced bystander effect. Antioxidants (Basel) (2021) 10(2):156. doi: 10.3390/antiox10020156 33494540 PMC7911176

[42] Folkard M Vojnovic B Prise KM Bowey AG Locke RJ Schettino G. A charged-particle microbeam: I. development of an experimental system for targeting cells individually with counted particles. Int J Radiat Biol (1997) 72(4):375–85. doi: 10.1080/095530097143158 9343103

[43] Folkard M Vojnovic B Hollis KJ Bowey AG Watts SJ Schettino G. A charged-particle microbeam: II. a single-particle micro-collimation and detection system. Int J Radiat Biol (1997) 72(4):387–95. doi: 10.1080/095530097143167 9343104

[44] Belyakov OV Malcolmson AM Folkard M Prise KM Michael BD. Direct evidence for a bystander effect of ionizing radiation in primary human fibroblasts. Br J Cancer. (2001) 84(5):674–9. doi: 10.1054/bjoc.2000.1665 PMC2363796 11237389

[45] Prise KM Belyakov OV Folkard M Michael BD. Studies of bystander effects in human fibroblasts using a charged particle microbeam. Int J Radiat Biol (1998) 74(6):793–8. doi: 10.1080/095530098141087 9881726

[46] Mosse I Marozik P Seymour C Mothersill C. The effect of melanin on the bystander effect in human keratinocytes. Mutat Res (2006) 597(1-2):133–7. doi: 10.1016/j.mrfmmm.2005.09.006 16412479

[47] Mothersill C Moran G McNeill F Gow MD Denbeigh J Prestwich W. A role for bioelectric effects in the induction of bystander signals by ionizing radiation? Dose Response. (2007) 5(3):214–29. doi: 10.2203/dose-response.06-011.Mothersill PMC2477697 18648606

[48] Ahmad SB McNeill FE Byun SH Prestwich WV Mothersill C Seymour C. Ultra-violet light emission from HPV-G cells irradiated with low let radiation from (90)Y; consequences for radiation induced bystander effects. Dose Response. (2013) 11(4):498–516. doi: 10.2203/dose-response.12-048.Ahmad 24298227 PMC3834743

[49] Kumar NM Gilula NB. The gap junction communication channel. Cell (1996) 84(3):381–8. doi: 10.1016/s0092-8674(00)81282-9 8608591

[50] Simon AM Goodenough DA. Diverse functions of vertebrate gap junctions. Trends Cell Biol (1998) 8(12):477–83. doi: 10.1016/s0962-8924(98)01372-5 9861669

[51] Azzam EI de Toledo SM Little JB. Direct evidence for the participation of gap junction-mediated intercellular communication in the transmission of damage signals from alpha -particle irradiated to nonirradiated cells. Proc Natl Acad Sci U S A. (2001) 98(2):473–8. doi: 10.1073/pnas.98.2.473 PMC14611 11149936

[52] Zhou H Suzuki M Randers-Pehrson G Vannais D Chen G Trosko JE. Radiation risk to low fluences of alpha particles may be greater than we thought. Proc Natl Acad Sci U.S.A. (2001) 98(25):14410–5. doi: 10.1073/pnas.251524798 PMC64695 11734643

[53] Persaud R Zhou H Baker SE Hei TK Hall EJ. Assessment of low linear energy transfer radiation-induced bystander mutagenesis in a three-dimensional culture model. Cancer Res (2005) 65(21):9876–82. doi: 10.1158/0008-5472.Can-04-2875 PMC4047718 16267011

[54] Cucinotta FA Chappell LJ. Non-targeted effects and the dose response for heavy ion tumor induction. Mutat Res (2010) 687(1-2):49–53. doi: 10.1016/j.mrfmmm.2010.01.012 20085778

[55] Azzam EI de Toledo SM Little JB. Oxidative metabolism, gap junctions and the ionizing radiation-induced bystander effect. Oncogene (2003) 22(45):7050–7. doi: 10.1038/sj.onc.1206961 14557810

[56] Imaizumi K Hasegawa Y Kawabe T Emi N Saito H Naruse K. Bystander tumoricidal effect and gap junctional communication in lung cancer cell lines. Am J Respir Cell Mol Biol (1998) 18(2):205–12. doi: 10.1165/ajrcmb.18.2.2821 9476907

[57] Yang H Asaad N Held KD. Medium-mediated intercellular communication is involved in bystander responses of X-ray-irradiated normal human fibroblasts. Oncogene (2005) 24(12):2096–103. doi: 10.1038/sj.onc.1208439 15688009

[58] Shu L Hollenberg PF. Identification of the cytochrome P450 isozymes involved in the metabolism of n-nitrosodipropyl-,N-nitrosodibutyl- and n-nitroso-n-butyl-n-propylamine. Carcinogenesis (1996) 17(4):839–48. doi: 10.1093/carcin/17.4.839 8625499

[59] Adler V Yin Z Tew KD Ronai Z. Role of redox potential and reactive oxygen species in stress signaling. Oncogene (1999) 18(45):6104–11. doi: 10.1038/sj.onc.1203128 10557101

[60] Spitz DR Sim JE Ridnour LA Galoforo SS Lee YJ. Glucose deprivation-induced oxidative stress in human tumor cells. a fundamental defect in metabolism? Ann N Y Acad Sci (2000) 899:349–62. doi: 10.1111/j.1749-6632.2000.tb06199.x 10863552

[61] Finkel T Holbrook NJ. Oxidants, oxidative stress and the biology of ageing. Nature (2000) 408(6809):239–47. doi: 10.1038/35041687 11089981

[62] Narayanan PK Goodwin EH Lehnert BE. Alpha particles initiate biological production of superoxide anions and hydrogen peroxide in human cells. Cancer Res (1997) 57(18):3963–71. 9307280

[63] Wu LJ Randers-Pehrson G Xu A Waldren CA Geard CR Yu Z. Targeted cytoplasmic irradiation with alpha particles induces mutations in mammalian cells. Proc Natl Acad Sci U S A. (1999) 96(9):4959–64. doi: 10.1073/pnas.96.9.4959 PMC21799 10220401

[64] Meister A Anderson ME. Glutathione. Annu Rev Biochem (1983) 52:711–60. doi: 10.1146/annurev.bi.52.070183.003431 6137189

[65] Azzam EI De Toledo SM Spitz DR Little JB. Oxidative metabolism modulates signal transduction and micronucleus formation in bystander cells from alpha-particle-irradiated normal human fibroblast cultures. Cancer Res (2002) 62(19):5436–42. 12359750

[66] Facoetti A Ballarini F Cherubini R Gerardi S Nano R Ottolenghi A. Gamma ray-induced bystander effect in tumour glioblastoma cells: a specific study on cell survival, cytokine release and cytokine receptors. Radiat Prot Dosimetry (2006) 122(1-4):271–4. doi: 10.1093/rpd/ncl431 17251249

[67] Facoetti A Mariotti L Ballarini F Bertolotti A Nano R Pasi F. Experimental and theoretical analysis of cytokine release for the study of radiation-induced bystander effect. Int J Radiat Biol (2009) 85(8):690–9. doi: 10.1080/09553000903020016 19637080

[68] Iyer R Lehnert BE. Effects of ionizing radiation in targeted and nontargeted cells. Arch Biochem Biophys (2000) 376(1):14–25. doi: 10.1006/abbi.1999.1684 10729186

[69] Assoian RK Fleurdelys BE Stevenson HC Miller PJ Madtes DK Raines EW. Expression and secretion of type beta transforming growth factor by activated human macrophages. Proc Natl Acad Sci U.S.A. (1987) 84(17):6020–4. doi: 10.1073/pnas.84.17.6020 PMC298999 2888109

[70] Ehrhart EJ Segarini P Tsang ML Carroll AG Barcellos-Hoff MH. Latent transforming growth factor beta1 activation in situ: quantitative and functional evidence after low-dose gamma-irradiation. FASEB J (1997) 11(12):991–1002. doi: 10.1096/fasebj.11.12.9337152 9337152

[71] Grotendorst GR Smale G Pencev D. Production of transforming growth factor beta by human peripheral blood monocytes and neutrophils. J Cell Physiol (1989) 140(2):396–402. doi: 10.1002/jcp.1041400226 2745570

[72] Lamarre J Vasudevan J Gonias SL. Plasmin cleaves betaglycan and releases a 60 kDa transforming growth factor-beta complex from the cell surface. Biochem J (1994) 302(Pt 1):199–205. doi: 10.1042/bj3020199 8068006 PMC1137210

[73] Lawrence DA. Transforming growth factor-beta: a general review. Eur Cytokine Netw (1996) 7(3):363–74. 8954178

[74] Munger JS Harpel JG Gleizes PE Mazzieri R Nunes I Rifkin DB. Latent transforming growth factor-beta: structural features and mechanisms of activation. Kidney Int (1997) 51(5):1376–82. doi: 10.1038/ki.1997.188 9150447

[75] Yahyapour R Amini P Rezapoor S Rezaeyan A Farhood B Cheki M. Targeting of inflammation for radiation protection and mitigation. Curr Mol Pharmacol (2018) 11(3):203–10. doi: 10.2174/1874467210666171108165641 29119941

[76] Klokov D Criswell T Leskov KS Araki S Mayo L Boothman DA. IR-inducible clusterin gene expression: a protein with potential roles in ionizing radiation-induced adaptive responses, genomic instability, and bystander effects. Mutat Res (2004) 568(1):97–110. doi: 10.1016/j.mrfmmm.2004.06.049 15530543

[77] Reddy KB Jin G Karode MC Harmony JA Howe PH. Transforming growth factor beta (TGF beta)-induced nuclear localization of apolipoprotein j/clusterin in epithelial cells. Biochemistry (1996) 35(19):6157–63. doi: 10.1021/bi952981b 8634259

[78] Jin G Howe PH. Transforming growth factor beta regulates clusterin gene expression via modulation of transcription factor c-fos. Eur J Biochem (1999) 263(2):534–42. doi: 10.1046/j.1432-1327.1999.00533.x 10406964

[79] Klokov D Leskov K Araki S Zou Y Goetz EM Luo X. Low dose IR-induced IGF-1-sCLU expression: a p53-repressed expression cascade that interferes with TGFβ1 signaling to confer a pro-survival bystander effect. Oncogene (2013) 32(4):479–90. doi: 10.1038/onc.2012.64 PMC3371099 22391565

[80] Hu W Xu S Yao B Hong M Wu X Pei H. MiR-663 inhibits radiation-induced bystander effects by targeting TGFB1 in a feedback mode. RNA Biol (2014) 11(9):1189–98. doi: 10.4161/rna.34345 PMC4615905 25483041

[81] Hu W Pei W Zhu L Nie J Pei H Zhang J. Microarray profiling of TGF-β1-Induced long non-coding RNA expression patterns in human lung bronchial epithelial BEAS-2B cells. Cell Physiol Biochem (2018) 50(6):2071–85. doi: 10.1159/000495052 30423581

[82] Matsumoto H Hayashi S Hatashita M Ohnishi K Shioura H Ohtsubo T. Induction of radioresistance by a nitric oxide-mediated bystander effect. Radiat Res (2001) 155(3):387–96. doi: 10.1667/0033-7587(2001)155[0387:Iorban]2.0.Co;2 11182788

[83] Leach JK Black SM Schmidt-Ullrich RK Mikkelsen RB. Activation of constitutive nitric-oxide synthase activity is an early signaling event induced by ionizing radiation. J Biol Chem (2002) 277(18):15400–6. doi: 10.1074/jbc.M110309200 11856735

[84] Shao C Furusawa Y Aoki M Matsumoto H Ando K. Nitric oxide-mediated bystander effect induced by heavy-ions in human salivary gland tumour cells. Int J Radiat Biol (2002) 78(9):837–44. doi: 10.1080/09553000210149786 12428924

[85] Shao C Stewart V Folkard M Michael BD Prise KM. Nitric oxide-mediated signaling in the bystander response of individually targeted glioma cells. Cancer Res (2003) 63(23):8437–42. 14679007

[86] Han W Chen S Yu KN Wu L. Nitric oxide mediated DNA double strand breaks induced in proliferating bystander cells after alpha-particle irradiation. Mutat Res (2010) 684(1-2):81–9. doi: 10.1016/j.mrfmmm.2009.12.004 20026341

[87] Zhou H Ivanov VN Gillespie J Geard CR Amundson SA Brenner DJ. Mechanism of radiation-induced bystander effect: role of the cyclooxygenase-2 signaling pathway. Proc Natl Acad Sci U S A. (2005) 102(41):14641–6. doi: 10.1073/pnas.0505473102 PMC1253564 16203985

[88] Lyng FM Maguire P McClean B Seymour C Mothersill C. The involvement of calcium and MAP kinase signaling pathways in the production of radiation-induced bystander effects. Radiat Res (2006) 165(4):400–9. doi: 10.1667/rr3527.1 16579652

[89] Prise KM O'Sullivan JM. Radiation-induced bystander signalling in cancer therapy. Nat Rev Cancer (2009) 9(5):351–60. doi: 10.1038/nrc2603 PMC2855954 19377507

[90] Lipp P Niggli E. Ratiometric confocal Ca(2+)-measurements with visible wavelength indicators in isolated cardiac myocytes. Cell Calcium (1993) 14(5):359–72. doi: 10.1016/0143-4160(93)90040-d 8519060

[91] Chen S Zhao Y Han W Zhao G Zhu L Wang J. Mitochondria-dependent signalling pathway are involved in the early process of radiation-induced bystander effects. Br J Cancer (2008) 98(11):1839–44. doi: 10.1038/sj.bjc.6604358 PMC2410123 18475304

[92] Bakkenist CJ Kastan MB. DNA Damage activates ATM through intermolecular autophosphorylation and dimer dissociation. Nature (2003) 421(6922):499–506. doi: 10.1038/nature01368 12556884

[93] Matsuoka S Ballif BA Smogorzewska A McDonald ER Hurov KE Luo J. ATM And ATR substrate analysis reveals extensive protein networks responsive to DNA damage. Science (2007) 316(5828):1160–6. doi: 10.1126/science.1140321 17525332

[94] Lee JH Paull TT. Activation and regulation of ATM kinase activity in response to DNA double-strand breaks. Oncogene (2007) 26(56):7741–8. doi: 10.1038/sj.onc.1210872 18066086

[95] Burdak-Rothkamm S Rothkamm K Prise KM. ATM Acts downstream of ATR in the DNA damage response signaling of bystander cells. Cancer Res (2008) 68(17):7059–65. doi: 10.1158/0008-5472.Can-08-0545 PMC2528059 18757420

[96] Rashi-Elkeles S Elkon R Weizman N Linhart C Amariglio N Sternberg G. Parallel induction of ATM-dependent pro- and antiapoptotic signals in response to ionizing radiation in murine lymphoid tissue. Oncogene (2006) 25(10):1584–92. doi: 10.1038/sj.onc.1209189 16314843

[97] Huxford T Huang DB Malek S Ghosh G. The crystal structure of the IkappaBalpha/NF-kappaB complex reveals mechanisms of NF-kappaB inactivation. Cell (1998) 95(6):759–70. doi: 10.1016/s0092-8674(00)81699-2 9865694

[98] Jacobs MD Harrison SC. Structure of an IkappaBalpha/NF-kappaB complex. Cell (1998) 95(6):749–58. doi: 10.1016/s0092-8674(00)81698-0 9865693

[99] Sen R Baltimore D. Inducibility of kappa immunoglobulin enhancer-binding protein nf-kappa b by a posttranslational mechanism. Cell (1986) 47(6):921–8. doi: 10.1016/0092-8674(86)90807-x 3096580

[100] Nelson G Kucheryavenko O Wordsworth J von Zglinicki T. The senescent bystander effect is caused by ROS-activated NF-κB signalling. Mech Ageing Dev (2018) 170:30–6. doi: 10.1016/j.mad.2017.08.005 PMC5861994 28837845

[101] Ivanov VN Zhou H Ghandhi SA Karasic TB Yaghoubian B Amundson SA. Radiation-induced bystander signaling pathways in human fibroblasts: a role for interleukin-33 in the signal transmission. Cell Signal (2010) 22(7):1076–87. doi: 10.1016/j.cellsig.2010.02.010 PMC2860693 20206688

[102] Ghandhi SA Yaghoubian B Amundson SA. Global gene expression analyses of bystander and alpha particle irradiated normal human lung fibroblasts: synchronous and differential responses. BMC Med Genomics (2008) 1:63. doi: 10.1186/1755-8794-1-63 19108712 PMC2627914

[103] Karin M. Nuclear factor-kappaB in cancer development and progression. Nature (2006) 441(7092):431–6. doi: 10.1038/nature04870 16724054

[104] Karin M Yamamoto Y Wang QM. The IKK NF-kappa b system: a treasure trove for drug development. Nat Rev Drug Discovery (2004) 3(1):17–26. doi: 10.1038/nrd1279 14708018

[105] Hei TK Zhou H Chai Y Ponnaiya B Ivanov VN. Radiation induced non-targeted response: mechanism and potential clinical implications. Curr Mol Pharmacol (2011) 4(2):96–105. doi: 10.2174/1874467211104020096 21143185 PMC3356574

[106] Schettino G Folkard M Michael BD Prise KM. Low-dose binary behavior of bystander cell killing after microbeam irradiation of a single cell with focused c(k) x rays. Radiat Res (2005) 163(3):332–6. doi: 10.1667/rr3319 15733040

[107] Sawant SG Randers-Pehrson G Geard CR Brenner DJ Hall EJ. The bystander effect in radiation oncogenesis: I. transformation in C3H 10T(1)/(2) cells in vitro can be initiated in the unirradiated neighbors of irradiated cells. Radiat Res (2001) 155(3):397–401. doi: 10.1667/0033-7587(2001)155[0397:Tbeiro]2.0.Co;2 11182789

[108] Wang R Coderre JA. A bystander effect in alpha-particle irradiations of human prostate tumor cells. Radiat Res (2005) 164(6):711–22. doi: 10.1667/3475.1 16296877

[109] Ponnaiya B Jenkins-Baker G Brenner DJ Hall EJ Randers-Pehrson G Geard CR. Biological responses in known bystander cells relative to known microbeam-irradiated cells. Radiat Res (2004) 162(4):426–32. doi: 10.1667/rr3236 15447040

[110] Shao C Furusawa Y Kobayashi Y Funayama T Wada S. Bystander effect induced by counted high-LET particles in confluent human fibroblasts: a mechanistic study. FASEB J (2003) 17(11):1422–7. doi: 10.1096/fj.02-1115com 12890695

[111] Autsavapromporn N Plante I Liu C Konishi T Usami N Funayama T. Genetic changes in progeny of bystander human fibroblasts after microbeam irradiation with X-rays, protons or carbon ions: the relevance to cancer risk. Int J Radiat Biol (2015) 91(1):62–70. doi: 10.3109/09553002.2014.950715 25084840

[112] Hall EJ Hei TK. Genomic instability and bystander effects induced by high-LET radiation. Oncogene (2003) 22(45):7034–42. doi: 10.1038/sj.onc.1206900 14557808

[113] Bishayee A Hill HZ Stein D Rao DV Howell RW. Free radical-initiated and gap junction-mediated bystander effect due to nonuniform distribution of incorporated radioactivity in a three-dimensional tissue culture model. Radiat Res (2001) 155(2):335–44. doi: 10.1667/0033-7587(2001)155[0335:friagj]2.0.co;2 PMC3495610 11175669

[114] Seymour CB Mothersill C. Relative contribution of bystander and targeted cell killing to the low-dose region of the radiation dose-response curve. Radiat Res (2000) 153(5 Pt 1):508–11. doi: 10.1667/0033-7587(2000)153[0508:rcobat]2.0.co;2 10790270

[115] Schettino G Folkard M Prise KM Vojnovic B Held KD Michael BD. Low-dose studies of bystander cell killing with targeted soft X rays. Radiat Res (2003) 160(5):505–11. doi: 10.1667/rr3060 14565833

[116] Liu Z Mothersill CE McNeill FE Lyng FM Byun SH Seymour CB. A dose threshold for a medium transfer bystander effect for a human skin cell line. Radiat Res (2006) 166(1 Pt 1):19–23. doi: 10.1667/rr3580.1 16808607

[117] Liu Z Lyun SH McNeill FE Mothersill CE Seymour CB Prestwich WV. Fluence and dose measurements for an accelerator neutron beam. Nucl Instruments Methods Phys Res Section B-Beam Interact Materials Atoms (2007) 263(1):326–8. doi: 10.1016/j.nimb.2007.04.256

[118] Coates PJ Rundle JK Lorimore SA Wright EG. Indirect macrophage responses to ionizing radiation: implications for genotype-dependent bystander signaling. Cancer Res (2008) 68(2):450–6. doi: 10.1158/0008-5472.Can-07-3050 18199539

[119] Kashino G Prise KM Schettino G Folkard M Vojnovic B Michael BD. Evidence for induction of DNA double strand breaks in the bystander response to targeted soft X-rays in CHO cells. Mutat Res (2004) 556(1-2):209–15. doi: 10.1016/j.mrfmmm.2004.08.009 15491649

[120] Lorimore SA Coates PJ Scobie GE Milne G Wright EG. Inflammatory-type responses after exposure to ionizing radiation *in vivo*: a mechanism for radiation-induced bystander effects? Oncogene (2001) 20(48):7085–95. doi: 10.1038/sj.onc.1204903 11704832

[121] Smith RW Seymour CB Moccia RD Hinton TG Mothersill CE. The induction of a radiation-induced bystander effect in fish transcends taxonomic group and trophic level. Int J Radiat Biol (2013) 89(4):225–33. doi: 10.3109/09553002.2013.754558 23206292

[122] Hatzi VI Laskaratou DA Mavragani IV Nikitaki Z Mangelis A Panayiotidis MI. Non-targeted radiation effects *in vivo*: a critical glance of the future in radiobiology. Cancer Lett (2015) 356(1):34–42. doi: 10.1016/j.canlet.2013.11.018 24333869

[123] Pant GS Kamada N. Chromosome aberrations in normal leukocytes induced by the plasma of exposed individuals. Hiroshima J Med Sci (1977) 26(2-3):149–54. 591380

[124] Emerit I Levy A Cernjavski L Arutyunyan R Oganesyan N Pogosian A. Transferable clastogenic activity in plasma from persons exposed as salvage personnel of the Chernobyl reactor. J Cancer Res Clin Oncol (1994) 120(9):558–61. doi: 10.1007/bf01221035 PMC12201545 8045922

[125] Marozik P Mothersill C Seymour CB Mosse I Melnov S. Bystander effects induced by serum from survivors of the Chernobyl accident. Exp Hematol (2007) 35(4 Suppl 1):55–63. doi: 10.1016/j.exphem.2007.01.029 17379088

[126] Zhou H Randers-Pehrson G Waldren CA Vannais D Hall EJ Hei TK. Induction of a bystander mutagenic effect of alpha particles in mammalian cells. Proc Natl Acad Sci U S A. (2000) 97(5):2099–104. doi: 10.1073/pnas.030420797 PMC15760 10681418

[127] Hu B Wu L Han W Zhang L Chen S Xu A. The time and spatial effects of bystander response in mammalian cells induced by low dose radiation. Carcinogenesis (2006) 27(2):245–51. doi: 10.1093/carcin/bgi224 16150894

[128] Ojima M Ban N Kai M. DNA Double-strand breaks induced by very low X-ray doses are largely due to bystander effects. Radiat Res (2008) 170(3):365–71. doi: 10.1667/rr1255.1 18763860

[129] Han W Wu L Hu B Zhang L Chen S Bao L. The early and initiation processes of radiation-induced bystander effects involved in the induction of DNA double strand breaks in non-irradiated cultures. Br J Radiol (2007) 80(Spec No 1):S7–12. doi: 10.1259/bjr/44550200 17704329

[130] Sedelnikova OA Rogakou EP Panyutin IG Bonner WM. Quantitative detection of (125) IdU-induced DNA double-strand breaks with gamma-H2AX antibody. Radiat Res (2002) 158(4):486–92. doi: 10.1667/0033-7587(2002)158[0486:Qdoiid]2.0.Co;2 12236816

[131] Hu B Han W Wu L Feng H Liu X Zhang L. *In situ* visualization of DSBs to assess the extranuclear/extracellular effects induced by low-dose alpha-particle irradiation. Radiat Res (2005) 164(3):286–91. doi: 10.1667/rr3415.1 16137201

[132] Kashino G Kondoh T Nariyama N Umetani K Ohigashi T Shinohara K. Induction of DNA double-strand breaks and cellular migration through bystander effects in cells irradiated with the slit-type microplanar beam of the spring-8 synchrotron. Int J Radiat Oncol Biol Phys (2009) 74(1):229–36. doi: 10.1016/j.ijrobp.2008.09.060 19362241

[133] Horn S Barnard S Rothkamm K. Gamma-H2AX-based dose estimation for whole and partial body radiation exposure. PloS One (2011) 6(9):e25113. doi: 10.1371/journal.pone.0025113 21966430 PMC3179476

[134] Han W Zhu L Jiang E Wang J Chen S Bao L. Elevated sodium chloride concentrations enhance the bystander effects induced by low dose alpha-particle irradiation. Mutat Res (2007) 624(1-2):124–31. doi: 10.1016/j.mrfmmm.2007.04.010 17560616

[135] Yang H Anzenberg V Held KD. The time dependence of bystander responses induced by iron-ion radiation in normal human skin fibroblasts. Radiat Res (2007) 168(3):292–8. doi: 10.1667/rr0864.1 17705636

[136] Sokolov MV Smilenov LB Hall EJ Panyutin IG Bonner WM Sedelnikova OA. Ionizing radiation induces DNA double-strand breaks in bystander primary human fibroblasts. Oncogene (2005) 24(49):7257–65. doi: 10.1038/sj.onc.1208886 16170376

[137] Ojima M Furutani A Ban N Kai M. Persistence of DNA double-strand breaks in normal human cells induced by radiation-induced bystander effect. Radiat Res (2011) 175(1):90–6. doi: 10.1667/rr2223.1 21175351

[138] Mothersill C Seymour CB. Cell-cell contact during gamma irradiation is not required to induce a bystander effect in normal human keratinocytes: evidence for release during irradiation of a signal controlling survival into the medium. Radiat Res (1998) 149(3):256–62. doi: 10.2307/3579958 9496888

[139] Frankenberg D Greif KD Giesen U. Radiation response of primary human skin fibroblasts and their bystander cells after exposure to counted particles at low and high LET. Int J Radiat Biol (2006) 82(1):59–67. doi: 10.1080/09553000600582979 16546904

[140] Shao C Aoki M Furusawa Y. Bystander effect on cell growth stimulation in neoplastic HSGc cells induced by heavy-ion irradiation. Radiat Environ Biophys (2003) 42(3):183–7. doi: 10.1007/s00411-003-0202-y 12920531

[141] Fournier C Barberet P Pouthier T Ritter S Fischer B Voss KO. No evidence for DNA and early cytogenetic damage in bystander cells after heavy-ion microirradiation at two facilities. Radiat Res (2009) 171(5):530–40. doi: 10.1667/rr1457.1 19580488

[142] Groesser T Cooper B Rydberg B. Lack of bystander effects from high-LET radiation for early cytogenetic end points. Radiat Res (2008) 170(6):794–802. doi: 10.1667/rr1458.1 19138042

[143] Koturbash I Rugo RE Hendricks CA Loree J Thibault B Kutanzi K. Irradiation induces DNA damage and modulates epigenetic effectors in distant bystander tissue *in vivo* . Oncogene (2006) 25(31):4267–75. doi: 10.1038/sj.onc.1209467 16532033

[144] Koturbash I Boyko A Rodriguez-Juarez R McDonald RJ Tryndyak VP Kovalchuk I. Role of epigenetic effectors in maintenance of the long-term persistent bystander effect in spleen *in vivo* . Carcinogenesis (2007) 28(8):1831–8. doi: 10.1093/carcin/bgm053 17347136

[145] Koturbash I Loree J Kutanzi K Koganow C Pogribny I Kovalchuk O. *In vivo* bystander effect: cranial X-irradiation leads to elevated DNA damage, altered cellular proliferation and apoptosis, and increased p53 levels in shielded spleen. Int J Radiat Oncol Biol Phys (2008) 70(2):554–62. doi: 10.1016/j.ijrobp.2007.09.039 18207032

[146] Koturbash I Zemp FJ Kutanzi K Luzhna L Loree J Kolb B. Sex-specific microRNAome deregulation in the shielded bystander spleen of cranially exposed mice. Cell Cycle (2008) 7(11):1658–67. doi: 10.4161/cc.7.11.5981 18560276

[147] Miralbell R Lomax A Cella L Schneider U. Potential reduction of the incidence of radiation-induced second cancers by using proton beams in the treatment of pediatric tumors. Int J Radiat Oncol Biol Phys (2002) 54(3):824–9. doi: 10.1016/s0360-3016(02)02982-6 12377335

[148] Autsavapromporn N Suzuki M Funayama T Usami N Plante I Yokota Y. Gap junction communication and the propagation of bystander effects induced by microbeam irradiation in human fibroblast cultures: the impact of radiation quality. Radiat Res (2013) 180(4):367–75. doi: 10.1667/rr3111.1 PMC4058832 23987132

[149] Mairs RJ Boyd M. Optimizing MIBG therapy of neuroendocrine tumors: preclinical evidence of dose maximization and synergy. Nucl Med Biol (2008) 35(Suppl 1):S9–20. doi: 10.1016/j.nucmedbio.2008.04.008 18707637

[150] McCluskey AG Boyd M Pimlott SL Babich JW Gaze MN Mairs RJ. Experimental treatment of neuroblastoma using I-131 meta-iodobenzylguanidine and topotecan in combination. Br J Radiol (2008) 81:S28–35. doi: 10.1259/bjr/27723093 18819996

[151] Mairs RJ Fullerton NE Zalutsky MR Boyd M. Targeted radiotherapy: microgray doses and the bystander effect. Dose Response. (2007) 5(3):204–13. doi: 10.2203/dose-response.07-002.Mairs PMC2477696 18648605

[152] Xue LY Butler NJ Makrigiorgos GM Adelstein SJ Kassis AI. Bystander effect produced by radiolabeled tumor cells in vivo. Proc Natl Acad Sci U S A. (2002) 99(21):13765–70. doi: 10.1073/pnas.182209699 PMC129772 12368480

[153] Kishikawa H Wang K Adelstein SJ Kassis AI. Inhibitory and stimulatory bystander effects are differentially induced by iodine-125 and iodine-123. Radiat Res (2006) 165(6):688–94. doi: 10.1667/rr3567.1 16802869

[154] Shusterman S Grant FD Lorenzen W Davis RT Laffin S Drubach LA. Iodine-131-labeled meta-iodobenzylguanidine therapy of children with neuroblastoma: program planning and initial experience. Semin Nucl Med (2011) 41(5):354–63. doi: 10.1053/j.semnuclmed.2011.06.001 21803185

[155] Strebhardt K Ullrich A. Paul Ehrlich's magic bullet concept: 100 years of progress. Nat Rev Cancer (2008) 8(6):473–80. doi: 10.1038/nrc2394 18469827

[156] Mavragani IV Nikitaki Z Souli MP Aziz A Nowsheen S Aziz K. Complex DNA damage: A route to radiation-induced genomic instability and carcinogenesis. Cancers (Basel) (2017) 9(7):91. doi: 10.3390/cancers9070091 28718816 PMC5532627

[157] Burdak-Rothkamm S Short SC Folkard M Rothkamm K Prise KM. ATR-dependent radiation-induced gamma H2AX foci in bystander primary human astrocytes and glioma cells. Oncogene (2007) 26(7):993–1002. doi: 10.1038/sj.onc.1209863 16909103

[158] Burdak-Rothkamm S Rothkamm K McClelland K Al Rashid ST Prise KM. BRCA1, FANCD2 and Chk1 are potential molecular targets for the modulation of a radiation-induced DNA damage response in bystander cells. Cancer Lett (2015) 356(2 Pt B):454–61. doi: 10.1016/j.canlet.2014.09.043 PMC4259524 25304378

[159] Lavin MF. ATM And the Mre11 complex combine to recognize and signal DNA double-strand breaks. Oncogene (2007) 26(56):7749–58. doi: 10.1038/sj.onc.1210880 18066087

[160] Tse AN Carvajal R Schwartz GK. Targeting checkpoint kinase 1 in cancer therapeutics. Clin Cancer Res (2007) 13(7):1955–60. doi: 10.1158/1078-0432.Ccr-06-2793 17404075

[161] Bahassi EM Ovesen JL Riesenberg AL Bernstein WZ Hasty PE Stambrook PJ. The checkpoint kinases Chk1 and Chk2 regulate the functional associations between hBRCA2 and Rad51 in response to DNA damage. Oncogene (2008) 27(28):3977–85. doi: 10.1038/onc.2008.17 18317453

[162] Dai Y Grant S. New insights into checkpoint kinase 1 in the DNA damage response signaling network. Clin Cancer Res (2010) 16(2):376–83. doi: 10.1158/1078-0432.Ccr-09-1029 PMC2939735 20068082

[163] Dahle J Kvam E Stokke T. Bystander effects in UV-induced genomic instability: antioxidants inhibit delayed mutagenesis induced by ultraviolet a and b radiation. J Carcinog. (2005) 4:11. doi: 10.1186/1477-3163-4-11 16091149 PMC1192812

[164] Burdak-Rothkamm S Smith A Lobachevsky P Martin R Prise KM. Radioprotection of targeted and bystander cells by methylproamine. Strahlenther Onkol. (2015) 191(3):248–55. doi: 10.1007/s00066-014-0751-9 PMC4338360 25245467

